# Eighteen Months of COVID-19 Pandemic Through the Lenses of Self or Others: A Meta-Analysis on Children and Adolescents’ Mental Health

**DOI:** 10.1007/s10566-022-09706-9

**Published:** 2022-08-29

**Authors:** Daniela Raccanello, Emmanuela Rocca, Giada Vicentini, Margherita Brondino

**Affiliations:** Department of Human Sciences, University of Verona, Lungadige Porta Vittoria 17, 37129 Verona, Italy

**Keywords:** COVID-19, Mental health, Self-report and other-report instruments, Children, Adolescents, Meta-analysis

## Abstract

**Background:**

The COVID-19 pandemic can have a serious impact on children and adolescents’ mental health. We focused on studies exploring its traumatic effects on young people in the first 18 months after that the pandemic was declared, distinguishing them also according to the type of informants (self-report and other-report instruments).

**Objective:**

We applied a meta-analytic approach to examine the prevalence of depression, anxiety, posttraumatic stress disorder, and psychological distress among children and adolescents during the COVID-19 pandemic, considering the moderating role of kind of disorder and/or symptom, type of instrument, and continent.

**Method:**

We used PsycINFO, PubMed, and Scopus databases to identify articles on the COVID-19 pandemic, applying the following filters: participants until 20 years of age, peer-review, English as publication language. Inclusion required investigating the occurrence of disorders and/or symptoms during the first 18 months of the pandemic. The search identified 26 publications.

**Results:**

The meta-analysis revealed that the pooled prevalence of psychological disorders and/or symptoms for children and adolescents, who were not affected by mental health disturbances before the outbreak of the COVID-19 pandemic, was .20, 95% CI [.16, .23]. Moreover, we found a moderating role of type of instrument: occurrence was higher for self-report compared to other-report instruments.

**Conclusions:**

The study presented an analysis of the psychological consequences for children and adolescents of the exposure to the COVID-19 pandemic, soliciting further research to identify factors underlying resilience. Notwithstanding limitations such as the small number of eligible articles and the fact that we did not examine the role of further characteristics of the studies (such as participants’ age or design), this meta-analysis is a first step for future research documenting the impact of such an unexpected and devastating disaster like the COVID-19 pandemic.

## Introduction

The COVID-19 pandemic is a biological natural disaster with a serious impact on both physical and mental health (EM-DAT, 2022; Jones et al., 2021). As many other disasters, pandemics can cause great suffering at the physical, biological, and social level, with dangerous consequences for individuals’ health and wellbeing (World Health Organization, 2022). Generally, children and adolescents exposed to a disaster are considered to be at risk, because of their heightened vulnerability. For this reason, they need special attention compared to adults during emergencies (Peek et al., 2018).

Disasters such as the COVID-19 pandemic have cascading and cumulative effects that pose many challenges to young people and their families (Masten & Motti-Stefanidi, 2020). In terms of mental health, among the traumatic consequences of the COVID-19 pandemic there can be an increase of psychological disorders and/or symptoms, such as depression, anxiety, posttraumatic stress disorder (PTSD), and sleep disorders in children and adolescents (Golberstein et al., 2020). The knowledge on their prevalence is paramount to develop and implement evidence-informed interventions to cope with the traumatic consequences of the pandemic and to foster their resilience, both during and after its occurrence. Therefore, we examined the literature on psychological disorders and/or symptoms, assessed through self-report or other-report instruments, in children and adolescents; we took into account studies published in the first 18 months of the COVID-19 pandemic, using a meta-analytical approach.

### Impact of COVID-19 on Children and Adolescents

On March 11, 2020, the World Health Organization (2022) declared that the spreading of the COVID-19 was a global pandemic. Many countries claimed a state of emergency, implementing strict public health measures. The safety measures taken, such as school closures, social distance, and indications on health protection behaviors, have had a strong impact on global mental health for children and adolescents (Ellis et al., 2020; Holmes et al., 2020). Recent literature confirmed that they are particularly exposed to the consequences of the COVID-19 pandemic from a psychological perspective (Cost et al., 2021; Kılınçel et al., 2020; Lavigne-Cerván et al., 2021; Ravens‑Sieberer et al., 2021; Tang et al., 2021). For example, lockdown periods have led to alterations of the sleep–wake rhythm, reduction of physical activity, and an excessive use of technology, as well as to increases of economic hardship, unemployment of many parents, and uncertainty concerning the future, causing generally high levels of psychological distress (Xie et al., 2020).

In the literature, many meta-analyses and systematic reviews well documented the psychological impact in children and adolescents of collective traumatic events, such as natural and technological disasters or violent acts. The PTSD is the most investigated consequence; however, some studies report the occurrence of a variety of disorders and/or symptoms, mainly associated with depression and anxiety, but also with other distress-related problems (Dimitry, 2012; Furr et al., 2010; Lai et al., 2014; Tang et al., 2014; Vibhakar et al., 2019; Wang et al., 2013). These results are in line with the already documented consequences on children and adolescents’ mental health of the COVID-19 pandemic, which is generally classified as a natural biological disaster (Cao et al., 2021; Islam et al., 2020; Ma et al., 2021; Mallik & Radwan, 2021; Shek et al., 2021; Zhou et al., 2020a). For example, during the pandemic the social isolation due to school closures and the requirement of physical distancing have brought to increases of depressive symptoms (Loades et al., 2020). Uncertainty, disruption in daily routines, and concern for one’s own and others’ health have caused a growth of generalized anxiety in youth (Courtney et al., 2020). A previous meta-analysis explored the psychological impact of the COVID-19 lockdown and quarantine measures examining studies published between December, 2019, and August, 2020. The authors identified 15 studies involving almost 23,000 children and adolescents. They showed that the prevalence of depression and anxiety was 34.5% and 41.7%, respectively; moreover, about 22.5% of the participants had also an intense fear of the COVID-19 (Panda et al., 2021). Racine et al. (2021) conducted a meta-analysis on the prevalence of depressive and anxiety symptoms with more than 80,000 children and adolescents involved in 29 studies published between January, 2020, and February, 2021. The results revealed that the depressive and anxiety symptoms varied, ranging from 21.2% to 29.7%, and from 17.2% to 24.4%, respectively. In particular, girls reported higher levels of anxiety and depression symptoms than boys. Moreover, depression symptoms were higher for adolescents than children.

However, in the two meta-analyses cited above scarce attention was paid to PTSD symptoms, mainly due to the fact that the COVID-19 pandemic is still in place. However, we know that PTSD is the most studied disorder in relation to other types of traumatic events (e.g., Wang et al., 2013) and it may be common among people with medical diseases (Salehi et al., 2021). Therefore, outbreaks of infectious diseases such as the COVID-19 can increase the risk of future PTSD symptoms among different populations, particularly involving patients who have been affected first-hand or who have seen a loved one—such as parents, siblings, or friends—getting sick (American Psychiatric Association, 2013).

Finally, several authors examined the psychological distress of young people during the COVID-19 pandemic, taking into account painful physical and mental symptoms that are not associated with a specific disorder, such as sleep disturbances, emotional symptoms, irritability, or social problems (Cost et al., 2021; Mallik & Radwan, 2021; Tamarit et al., 2020; Wang et al., 2021b). For example, a meta-analysis by Sharma et al. (2021) reported that the prevalence of sleep disturbances in children and adolescents during the pandemic was 54%. In addition, some authors revealed a significant increase in emotional, social, and behavioral problems (Cost et al., 2021; Liu et al., 2021a; Wang et al., 2021a). For example, Liu et al. (2021a) investigated the prevalence of conduct problems and emotional symptoms, highlighting a significant increase during the pandemic.

The occurrence of disorders and/or symptoms of depression, anxiety, PTSD, and psychological distress can vary according to different factors, such as the type of instrument utilized to measure them and the geographic area in which the study was conducted. Concerning the instruments, we can distinguish between self-report and other-report measures of disorders and/or symptoms. Self-report measures enable to emphasize the personal perspective of the respondent and have the advantage of permitting to investigate inner states that are difficult to observe from the outside (Pekrun & Bühner, 2014). Other-report measures consider the external perspective of valid informants (usually the parents) and are particularly useful when the children are too young or when the social conditions make it difficult to reach children and/or adolescents directly (Masten & Osofsky, 2010), as occurred during the COVID-19 pandemic. In some meta-analyses concerning post-disaster mental health in children and adolescents, the type of instrument resulted a significant moderator. For example, in a meta-analysis by Furr et al. (2010) about PTSD after natural and technological disasters, the symptoms were more frequent when the measures were self-report rather than other-report. Another meta-analysis on the prevalence of depression after trauma (Vibhakar et al., 2019) showed that the negative symptoms due to the exposure were deeper when identified through diagnostic interviews rather than self-report measures. In this case, however, the informants differed also for their specific competence on mental health issues. Rubens et al. (2018), in a meta-analysis on the psychological impact of natural disasters, found that the association between disaster exposure and externalizing problems was stronger when the instruments were other-report. These findings give some suggestions about the characteristics of self-report and other-report instruments when informants are lay people. On the one hand, internalizing disorders and/or symptoms (such as depression, anxiety, and PTSD) can be better described using self-report instruments, which permit a privileged perspective about the interior states. On the other hand, externalizing ones (such as behavioral problems) can be better investigated through other-report measures for their external nature.

Concerning the geographic area, the COVID-19 pandemic is a worldwide phenomenon and its effects have affected every continent. However, the social and medical responses to the spreading of the coronavirus and the disease that it causes have been diverse in different countries, with possible variations in the consequences both for children and adolescents’ physical and mental health (for an example concerning adults, see Raccanello et al., 2022a). In some cases, previous meta-analyses on the psychological effects of disasters revealed a moderating effect of the involved countries, based mainly on socio-economic differences: the disorders and/or symptoms were stronger in low/medium-income countries compared to high-income countries (Rubens et al., 2018; Vibhakar et al., 2019).

In summary, the psychological literature suggests that the exposure to a traumatic event has negative consequences for people’s mental health. Compared to adults, children and adolescents are particularly vulnerable because of their different level of cognitive and emotional development. In the current emergency situation caused by the COVID-19, the prevention of the spreading of the virus has been implemented by measures such as mass quarantine, school closures, and social distancing. Within the pandemic framework, high levels of stress can emerge given the sudden and important changes in children and adolescents’ daily life (Brooks et al., 2020). To our knowledge, no previous study has examined together, through a meta-analytic approach, different psychological disorders and/or symptoms such as depression, anxiety, PTSD, and psychological distress due to the COVID-19 pandemic, in children and adolescents, differentiating self-report and other-report instruments. Understanding the psychological impact of the COVID-19 pandemic on this population would provide a theoretical basis for designing timely interventions to protect young people from such events in the future (Pappa et al., 2020; Vicentini et al., 2020). Given the large number of factors associated with children and adolescents’ responses to a disaster (Masten & Osofsky, 2010), it is of paramount relevance to identify how children and adolescents are reacting to foster their resilience (Masten & Motti-Stefanidi, 2020).

### Current Study and Research Questions

The aim of this meta-analysis was to examine the psychological impact of the COVID-19 pandemic on children and adolescents’ mental health, taking into account the occurrence of psychological disorders and/or symptoms, such as depression, anxiety, PTSD, and psychological distress. We examined studies published in the first 18 months after that the World Health Organization declared the pandemic in March 2020. We also investigated the moderating role of some factors.

In line with previous systematic reviews and meta-analyses on the psychological effects of the COVID-19 pandemic for children and adolescents (Ma et al., 2021; Panda et al., 2021; Racine et al., 2021; Sharma et al., 2021) and research on the variety of mental health consequences of the pandemic (Cost et al., 2021; Islam et al., 2020; Lavigne-Cerván et al., 2021; Mallik & Radwan, 2021; Ravens-Sieberer et al., 2021; Tamarit et al., 2020; Tang et al., 2021; Wang et al., 2021b), we formulated two research questions, focusing on studies about psychological disorders and/or symptoms in children and adolescents, concerning the first 18 months of the COVID-19 pandemic. Which was the prevalence of depression, anxiety, PTSD, and psychological distress among children and adolescents not affected by them before the outbreak of the COVID-19 pandemic (Research Question 1)? We also investigated the moderating role of factors such as the kind of disorder and/or symptom (depression, anxiety, PTSD, and psychological distress), the instrument used to assess disorders and/or symptoms (self-report and other-report), and the continent (North America, Asia, and Europe): how did these factors influence the prevalence of psychological disorders and/or symptoms (Research Question 2)?

This meta-analysis is part of a larger project aimed at fostering emotional preparedness of children, adolescents, and adults to cope with natural and technological disasters and violent acts (HEMOT project, Helmet for EMOTions, https://www.hemot.eu; Raccanello et al., 2020a, 2020b, 2020c, 2021, 2022b; Vicentini et al., 2020).

## Method

### Literature Search and Search Results

We conducted systematic searches in three databases during September 2021: PsycINFO, PubMed, and Scopus. We decided to focus on PsycINFO and PubMed as they are among the most authoritative databases for conducting meta-analyses in mental health research (Cuijpers, 2016). In addition, following guidelines suggesting not to confine reviews to one or two databases (Cheung & Vijayakumar, 2016; Lemeshow et al., 2005), we decided to examine also a citation database like Scopus to find other relevant articles, in line with previous experiences (e.g., Filatova et al., 2017; Hendriks et al., 2018). We used the following search terms: “COVID-19” AND “children and adolescents” AND “mental health”. Concerning inclusion criteria, we considered studies which: (a) involved participants until 20 years of age; (b) included the assessment of at least one measure of psychological disorders and/or symptoms; (d) reported the occurrence of disorders and/or symptoms so that effect sizes (*ES*) could be calculated; (e) analyzed data that were collected during the COVID-19 pandemic; (f) were written in English. We excluded publications reporting reviews, discussions, single-case studies, and qualitative studies. Moreover, we excluded those studies whose participants suffered from physical or mental illness prior to the COVID-19 pandemic and those studies which did not include the statistical indexes necessary as inputs for a meta-analysis.

**Fig. 1:**
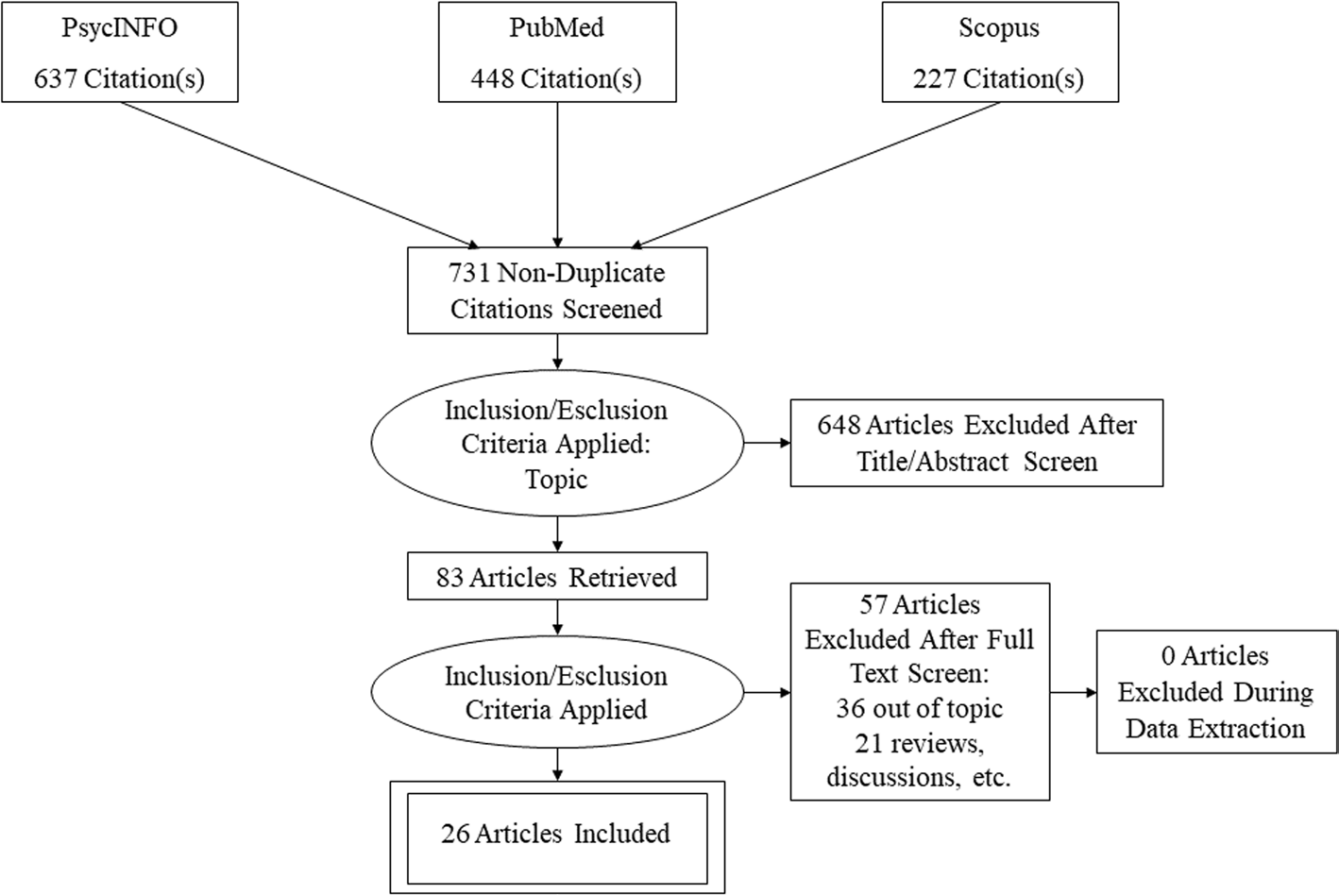
PRISMA diagram (Moher et al., 2009)

The initial search identified a total of 1163 works published between the outbreak of the pandemic and September 30, 2021. Four-hundred and thirty-eight publications were indexed in PsycINFO, 448 were indexed in PubMed, and 277 were indexed in Scopus. As a first step, we removed 432 duplicates from this initial set, i.e., the same publications downloaded in different searches. Then, we screened the 731 selected publications. As a second step, we read all the titles and abstracts and included only the publications pertinent in terms of topic—i.e., respecting the inclusion criteria—for a total of 83. As a third step, we read each article, and this led us to exclude 36 publications because they were off topic, and 21 because they reported reviews, discussions, single-case studies, and qualitative studies. This last step of the selection process was conducted by two independent judges; the reliability was 100%. No publications were excluded after the discussion between judges. Thus, the search identified a selection of 26 publications. We report in the PRISMA diagram (Fig. 1) the results of the selection process (Moher et al., 2009).

For ethical issues, we adhered to the recommendations of the American Psychological Association.

### Coding and Reliability

We reviewed and coded the eligible studies for several variables. We coded measures of psychological disorders and/or symptoms, in terms of occurrence of depression, anxiety, PTSD, and psychological distress. We also coded the type of instrument (distinguishing self-report and other-report instruments) and the continent (North America, Asia, and Europe). The moderating effect of age was not examined, because in most studies children and adolescents were not separated. For an overview of the included studies, see Table 1.

**Table 1:** Overview of the selected studies

References	Journal	Country (continent)	Time from data collection	*N* (% F)	Age (range or mean age in years)	Kind of psychological disorder and/or symptom	Category of psychological disorder/symptom	Instruments for psychological disorders/symptoms	Type of instrument
Cao et al. (2021)	Journal of Affective Disorders	China (Asia)	March 2020	11,681 (39.9%)	12–18	Depression symptoms Anxiety symptoms	Depression Anxiety	Patient Health Questionnaire for Depression (PHQ-9) Generalized Anxiety Disorder Scale (GAD-7)	Self-report
Chen et al. (2021)	Translational Psychiatry	China (Asia)	February 2020 April 2020	9552 (52.1%) 3886 (53.8%)	11–20	Depression Anxiety	Depression Anxiety	Chinese version of the Center for Epidemiological Studies-Depression Scale (CES-D) Generalized Anxiety Disorder Scale (GAD-7)	Self-report
Cost et al. (2021)	European Child & Adolescent Psychiatry	Canada (America)	April–June 2020	763 (48.1%)	10–18	Depression Anxiety Irritability	Depression Anxiety Psychological distress	Coronavirus Health and Impact Survey (CRISIS)	Other-report
Duan et al. (2020)	Journal of Affective Disorders	China (Asia)	January 2020	3631 (49.9%)	7–18	Depression symptoms	Depression	Child Depression Inventory (CDI)	Self-report
Ellis et al. (2020)	Canadian Journal of Behavioural Science	Canada (America)	April 2020	1054 (76.4%)	14–18	Depression Fear	Depression Anxiety	COVID-19 stress (adapted from Swine Flu Anxiety Scale)	Self-report
Gladstone et al. (2021)	Child Psychiatry & Human Development	USA (America)	May 2020	228 (53%)	12–18	Depression symptoms	Depression	Patient Health Questionnaire‑Adolescent (PHQ‑A)	Self-report
Hu et al. (2021)	Children and Youth Services Review	China (Asia)	January–March 2020	2090 (62.4%)	12–18	Fear PTSD	Anxiety PTSD	COVID-19 related exposure Children’s Revised Impact of Event Scale (CRIES)	Self-report
Islam et al. (2020)	Journal of Affective Disorders	Bangladesh (Asia)	March–April 2020	306	13–20	Anxiety	Anxiety	Generalized Anxiety Disorder Scale (GAD-7)	Self-report
Kılınçel et al. (2020)	Asia–Pacific Psychiatry	Turkey	March 2020	745 (69.5%)	12–18	Anxiety	Anxiety	State Anxiety Inventory (STAI-S)	Self-report
Lavigne-Cerván et al. (2021)	Frontiers in Psychology	Spain (Europe)	April 2020	1028 (46.5%)	6–18	Anxiety	Anxiety	State-Trait Anxiety Inventory for Children (STAIC)	Other-report
Li et al. (2021)	European Child & Adolescent Psychiatry	Australia	June–August 2020	760 (72%)	12–18	Anxiety Psychological distress	Anxiety Psychological distress	Three-item Body Preoccupation Scale of the Illness Attitude Scales Kessler-6 (K6)	Self-report
Liu et al. (2021a)	Journal of Affective Disorders	China (Asia)	February–March 2020	1784 (44.1%)	7–12	Emotional symptoms	Psychological distress	Strengths and Difficulties Questionnaire (SDQ)	Self-report
Liu et al. (2021b)	Journal of Affective Disorders	China (Asia)	June 2020	5175 (48.4%)	9–18	Depression Anxiety	Depression Anxiety	Chinese version of the Patient Health Questionnaire (PHQ-9) Chinese version of Generalized Anxiety Disorder (GAD-7)	Self-report
Ma et al. (2021)	BMC Pediatrics	China (Asia)	April 2020	668 (49.7%)	7–15	Depression PTSD	Depression PTSD	Short Mood and Feelings Questionnaire (SMFQ-P) Impact of Events Scale-Revised (IES-R)	Other-report
Mallik and Radwan (2021)	Asian Journal of Psychiatry	Bangladesh (Asia)	March 2020	552	4–17	Emotional disorder	Psychological distress	Bangla Strengths and Difficulties Questionnaire (SDQ)	Other-report
Ravens-Sieberer et al. (2021)	European Child & Adolescent Psychiatry	Germany (Europe)	May–June 2020	1040 (51.1%)	11–17	Anxiety Mental health problems	Anxiety Psychological distress	German version of the Screen for Child Anxiety Related Disorders (SCARED) Strengths and Difficulties Questionnaire (SDQ)	Self-report
Shek et al. (2021)	Journal of Adolescent Health	China (Asia)	June–July 2020	4981 (48.5%)	11–20	PTSD	PTSD	Children’s Revised Impact of Event Scale (CRIES-13)	Self-report
Tamarit et al. (2020)	Revista de Psicología Clínica con Niños y Adolescentes	Spain (Europe)	May–July 2020	523 (63.1%)	13–17	Depression Anxiety Stress	Depression Anxiety Psychological distress	Depression, Anxiety and Stress Scale (DASS-21)	Self-report
Tang et al. (2021)	Journal of Affective Disorders	China (Asia)	March 2020	4342 (49%)	6–17	Depression Anxiety Stress	Depression Anxiety Psychological distress	Chinese version of the Depression Anxiety Stress Scale-21 (DASS-21)	Self-report
Walters et al. (2021)	School Psychology	USA (America)	November 2020	309	10–16	Depression	Depression	Center for Epidemiological Studies Depression scale (CES-D)	Self-report
Wang et al. (2021a)	Journal of Affective Disorders	China (Asia)	May–July 2020	12,186 (47.8%)	6–16	Depression Social problems	Depression Psychological distress	Chinese version of the Achenbach Child Behaviour Checklist (CBCL)	Other-report
Wang et al. (2021b)	Globalization and Health	China (Asia)	April–May 2020	6435 (50.2%)	12–18	Depression	Depression	Children’s Depression Inventory (CDI)	Self-report
Zhang et al. (2020)	JAMA Network Open	China (Asia)	May 2020	1241 (40.7%)	9–16	Depression Anxiety	Depression Anxiety	Mood and Feelings Questionnaire (MFQ) MacArthur Health & Behavior Questionnaire	Self-report
Zhou et al. (2020a)	European Child & Adolescent Psychiatry	China (Asia)	March 2020	8097	12–18	Depressive symptoms Anxiety symptoms	Depression Anxiety	Patient Health Questionnaire (PHQ-9)	Self-report
Zhou et al. (2020b)	Globalization and Health	China (Asia)	February 2020	4805 (100%)	11–18	Depression	Depression	Chinese version of the Center for Epidemiological Studies Depression Scale (CES-D)	Self-report

#### Measures

Several authors explored the incidence of psychological disorders and/or symptoms during the COVID-19 pandemic on children and adolescents. We coded their occurrence, distinguishing them in four categories: depression (e.g., Cao et al., 2021; Tang et al., 2021), anxiety (e.g., Islam et al., 2020; Tamarit et al., 2020), PTSD (e.g., Hu et al., 2021; Shek et al., 2021), and psychological distress (e.g., Cost et al., 2021; Mallik & Radwan, 2021). A first judge coded all the selected articles for each psychological disorder and/or symptom. A second judge coded 30% of them for reliability. The Cohen’s ĸ was .98.

We also coded whether the instrument used to assess disorders and/or symptoms was self-report or other-report, and the continent where the participants were recruited (North America, Asia, and Europe). A first judge coded all the articles for both variables; a second judge coded 30% of them. The Cohen’s ĸ was 1.00 and .98, respectively.

### Data Analysis

We conducted a meta-analysis to examine the prevalence of psychological disorders and/or symptoms among children and adolescents during the COVID-19 pandemic. We extracted from each study the data on the number of participants affected by the disorder and/or symptom and the total number of each sample. We conducted the statistical analyses using the Metafor package of R, Version 4.0.5 (R Core Team, 2020). At first, the application of transformations to proportional data was needed to assure that the transformed proportions had a normal distribution, and therefore to assure an adequate estimate of the pooled proportion and increase the validity of the related statistical analyses. We followed the suggestions of Wang (2018) to apply Freeman-Tukey double arcsine transformation (Freeman & Tukey, 1950; Miller, 1978). In particular, we used the transformed proportions as effect sizes and the inverse of the variance of the transformed proportions as study weights for all the analyses. Then, the double arcsine transformations were converted back into proportions to report the results.

We calculated the prevalence estimates of disorders by pooling the study-specific estimates using a random-effects meta-analysis which takes both within- and between-study variances into account (Borenstein et al., 2010). Because in some cases we extracted more than one proportion from the same study, in our dataset there were interdependent effect sizes. Therefore, we utilized the multilevel approach to deal with interdependency (Assink & Wibbelink, 2016). In particular, to take into account the interdependency within studies we used the “rma.mv” function of the Metafor package, which assigns the same random effects to the transformed proportions with the same value of the grouping variable. We also analyzed the intra-class correlation (ICC) to verify whether the multilevel approach was appropriate—i.e., it is adequate whether the ICC values are higher than .05 (LeBreton & Senter, 2008).

Following the suggestion of Van den Noortgate and Onghena (2003), we used a two-step procedure. First, we ran a traditional random-effects meta-analysis: we evaluated the main effects, performed the influence analyses, and checked the publication bias. Second, we ran a multilevel mixed-effects meta-analysis to examine the role of the moderators. We checked whether the results of the traditional random-effects model differed from the results of the multilevel mixed-effects model, and we found that they were not substantially different.

For the multilevel mixed-effects models, we chose to use the restricted maximum-likelihood estimation method, because it seemed more appropriate for considering non-independent sampling errors due to the presence of multiple effects in the studies (Borenstein, 2009). We used Cochran’s heterogeneity statistic (*Q*) to test if the effect sizes of different studies were similar or not. A significant value of *Q* means that there is heterogeneity between the effect sizes. We used the *I*^*2*^ statistic to assess the level of heterogeneity. It measures the proportion of total variance due to the variability between the studies. We have low heterogeneity if the value of the statistic is between 1 and 49, medium if the value is between 50 and 74, and high if the value is between 75 and 100. We assessed the role of each moderator with mixed-effect models, considering the dependence of effect sizes through multilevel modelling (level 1 = effect sizes; level 2 = study). We conducted a test to evaluate the possible moderating effect of one or more variables included in the model. In this test, the null hypothesis was that all the regression coefficients were equal to zero, while the alternative hypothesis was that at least one of the regression coefficients was not equal to zero.

We examined the moderating role of the kind of disorder and/or symptom (depression, anxiety, PTSD, and psychological distress), the type of instrument used to assess disorders and/or symptoms (self-report and other-report), and the continent (North America, Asia, and Europe). We excluded those studies that did not have information on each moderator in the corresponding analysis. We assessed the potential publication biases using the trim and fill approach of Duval and Tweedie (2000). This approach estimates the number of studies missing from a meta-analysis by eliminating those studies that create patterns of asymmetry, and adding new data estimated on the initial sample to generate a symmetrical distribution of effect sizes. The output of this analysis is a funnel plot that is designed using the effect size against the standard error for each study.

## Results

### Prevalence of Psychological Symptoms and/or Disorders in Children and Adolescents

Initially, we analyzed the data concerning the proportions of children and adolescents affected by psychological disorders and/or symptoms reported in the studies included in the meta-analysis. Their incidence ranged from .07 to .67. Then, we transformed the proportions applying the Freeman-Tukey double arcsine transformation and we ran a first random-effects model. This model, *k* = 47, *n* = 155.282, estimated a pooled incidence of psychological disorders and/or symptoms equal to .21, 95% CI [.17, .26], *SE* = .03. The effect sizes were heterogeneous, *Q*(46) = 22,862.65, *p* < .001, and the proportion of total variance due to the variability between the studies was very high, *I*^*2*^ = 99.76%.

**Fig. 2:**
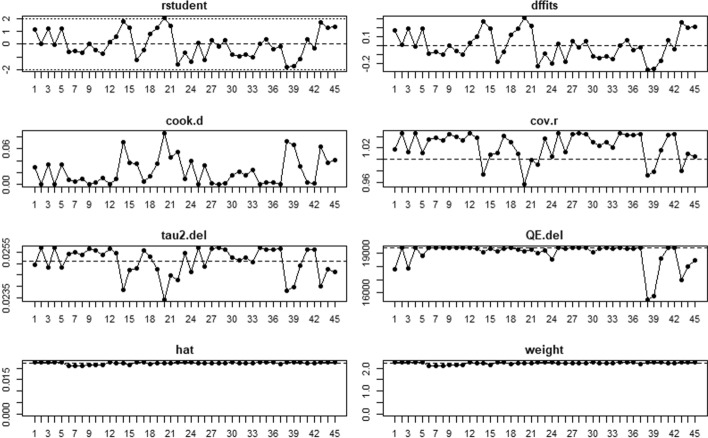
Influence analysis of psychological disorders and/or symptoms for each study

**Fig. 3:**
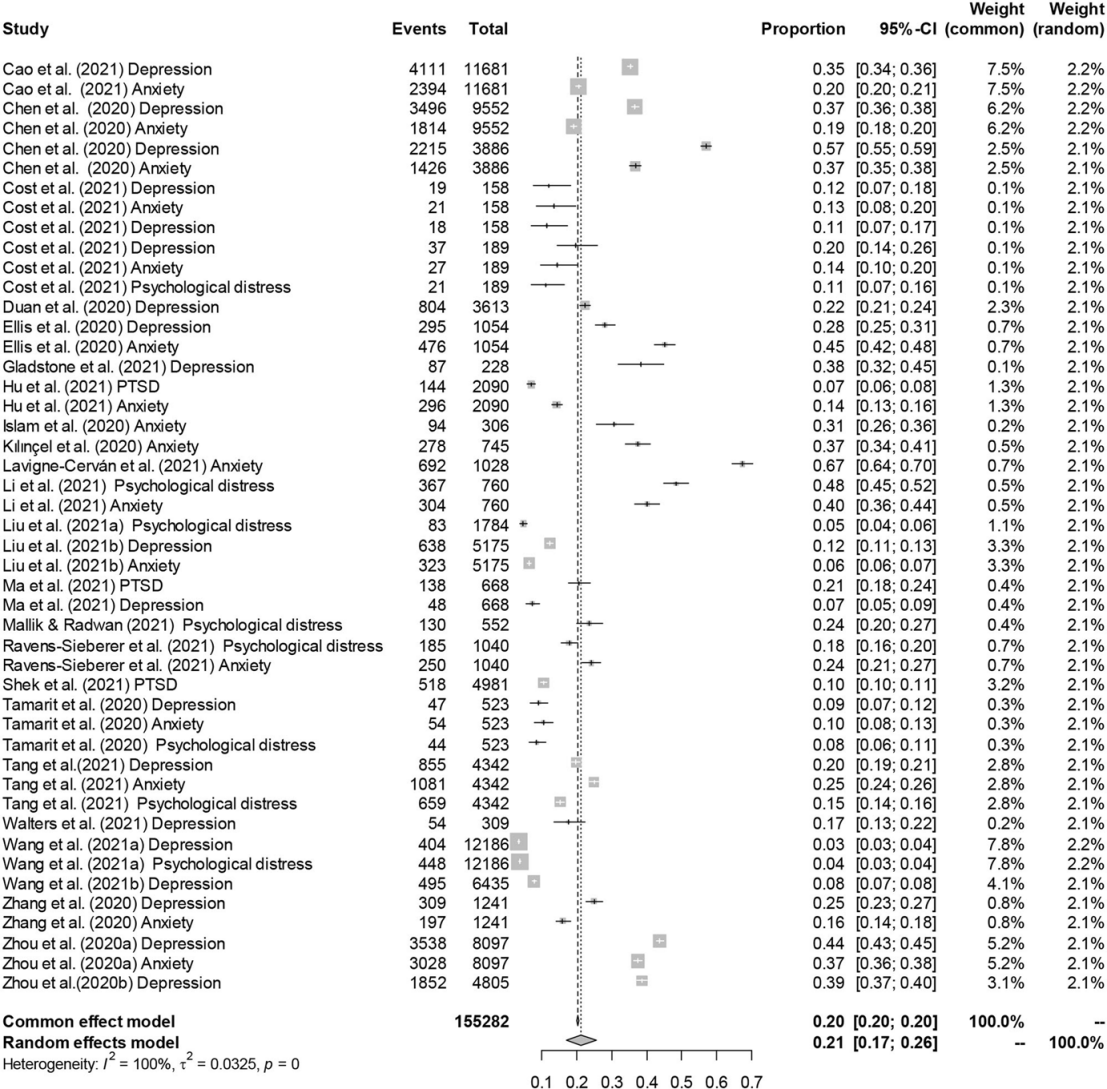
Forest plot of psychological disorders and/or symptoms. For each study there were one or more kinds of disorder and/or symptom (see Table 1)

Considering the forest plot, we identified two potential outlying effect sizes. We examined them further to determine whether they were really influential to the overall effect size. Following Viechtbauer and Cheung’s (2010) suggestions, we analyzed the outlying effect sizes (Chen et al., 2021; Lavigne-Cerván et al., 2021) by screening for externally standardized residuals and we saw that they were greater than 2. Lavigne-Cerván et al. (2021) reported a proportion equal to .67 and Chen et al. (2021) a proportion equal to .57, both very far from the estimated summary proportion. However, to detect influential studies we also considered other case deletion diagnostics, such as Cook’s distances, the influence of individual studies on heterogeneity (*Q* statistic), and leave-one-out estimates for the amount of heterogeneity, and they confirmed these studies as outliers (Fig. 2). Consequently, we decided to leave them out and we ran a second random-effects model, *k* = 45, *n* = 150.37. The estimated pooled incidence of psychological disorders and/or symptoms slightly changed, and it was equal to .20, 95% CI [.16, .23], *SE* = .02. The new analysis confirmed a high heterogeneity, *Q*(44) = 19,408.58, *p* < .001, and again the proportion of total variance due to the variability between the studies was very high, *I*^2^ = 99.69%. The forest plot is shown in Fig. 3.

**Fig. 4:**
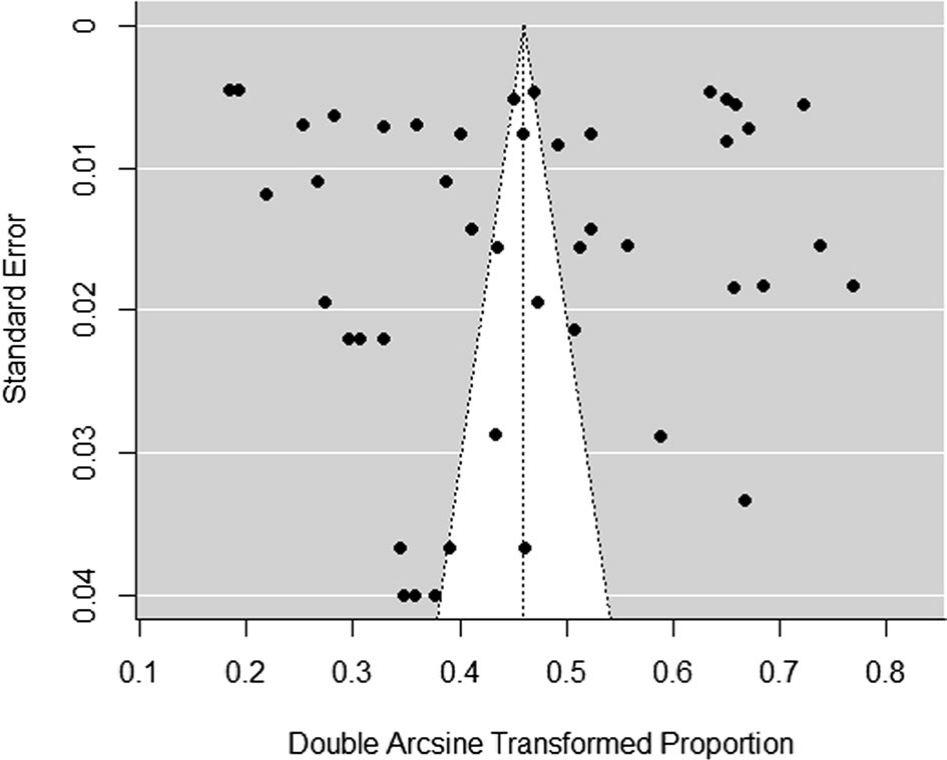
Funnel plot of psychological disorders and/or symptoms. For each study there were one or more kinds of disorder and/or symptom (see Table 1)

We then explored the potential presence of a publication bias, applying the trim and fill approach. The test was not significant, suggesting that there was no presence of publication biases (see funnel plot in Fig. 4).

After that, we conducted a multilevel random-effects model on the final sample of studies to compare the obtained estimated pooled prevalence of psychological disorders and/or symptoms with that of the second model. The summary proportion estimated through the multilevel model was actually close to that of the second model, being it equal to .20, 95% CI [.16, .24], *SE* = .025 (Van den Noortgate & Onghena, 2003). We also calculated the ICC, that was .31, confirming the adequacy of adopting multilevel analyses.

### Moderation of Kind of Disorder and/or Symptom, Type of Instrument, and Continent

The subsequent step was to run multilevel mixed-effects models to assess the role of potential moderators in explaining the high heterogeneity between the effect sizes. We examined the role of kind of disorder and/or symptom, the type of instrument, and the continent. Only the second moderator seemed to explain the variability between the proportions.

After that the type of instrument has been included to the meta-analytic model, there was still a significant unexplained variance left between all effect sizes in the data set, *Q*_*RESIDUAL*_(43) = 11,513.28, *p* < .001. However, the test highlighted a moderating role of this variable, *F*(1, 43) = 6.25, *p* = .016. The combined prevalence of children and adolescents with psychological disorders and/or symptoms using self-report instrument was equal to .22, 95% CI [.18, .27], while it was lower and equal to .11, 95% CI [.06, .19], when other-report instruments were used.

## Discussion

This meta-analysis aimed at examining the psychological impact of the COVID-19 pandemic on children and adolescents’ mental health, taking into account studies published during the first 18 months after that the World Health Organization declared the pandemic, on 11 March, 2020. We extended previous works focusing both on a longer period and considering a wider variety of psychological disorders and/or symptoms, particularly the PTSD, frequently neglected by previous meta-analyses on this pandemic. Beyond being interested in documenting the prevalence of different disorders and/or symptoms, we examined some characteristics of the instruments used to assess them, i.e., self-report or other-report. In doing so, we sought to extend the corpus of knowledge that explored differences in the ways in which respondents different from children or adolescents report information on their mental health (Furr et al., 2010; Rubens et al., 2018; Vibhakar et al., 2019). Therefore, we focused on two different lenses that can be used to observe the traumatic consequences of a disaster such as a pandemic. Finally, given the widespread diffusion of the COVID-19, our analyses aimed at describing possible differences in children and adolescents’ reactions that could be linked to their geographic provenience.

In accordance with the existing literature on the psychological effects of the COVID-19 pandemic (Ma et al., 2021; Panda et al., 2021; Racine et al., 2021; Sharma et al., 2021), our results allowed us to describe the prevalence of psychological disorders and/or symptoms during the COVID-19 pandemic for children and adolescents not affected by disorders before its outbreak (Research Question 1). The state of emergency included both the danger to physical health and a set of measures taken to prevent infection (e.g., school closure, social distance, and lockdown periods), which probably combined their effects leading, in some cases, to the development of disorders and/or symptoms such as depression, anxiety, PTSD, and psychological distress in this population. In particular, this meta-analysis revealed a rate of 20% of occurrence of such disturbances in children and adolescents not affected by them before the outbreak of the COVID-19 pandemic.

In addition, we examined the moderating role of three factors, i.e., the kind of disorder and/or symptom, the type of instrument, and the continent (Research Question 2). On the one hand, the results suggested that only one of these moderators explained the variability between the proportions, i.e., the type of instrument. In line with previous studies (e.g., Furr et al., 2010), we found that the prevalence of children and adolescents with psychological disorders was higher when it was assessed through self-report instruments rather than other-report instruments. In previous research, this seemed to happen in case of internalizing problems. In other terms, it could be difficult for caregivers or relevant informants without specific diagnostic competences to identify adequately psychological disturbances with such characteristics. Given the need to resort to other-report instruments in some specific contexts (Masten & Osofsky, 2010), this result should be taken into account deeper by future research. Nevertheless, a meta-analysis about quite a large number of studies on disasters such as terrorism, tsunamis, and hurricanes with children and adolescents indicated that self-report measures were more frequent than other report-measures (Pfefferbaum et al., 2013). Moreover, according to Grolnick et al. (2018), it is relevant to consider that children and adolescents are more reliable informants, compared to other persons, concerning their experiences, perceptions, and emotions (Myers & Winters, 2002). Therefore, whenever it is possible, self-report instruments should be privileged.

On the other end, nor the kind of disorder and/or symptom neither the continent resulted as significant moderators. We could speculate that this finding strongly support the pervasive traumatic effects of the pandemic independently of both specific disturbances and geographical areas. However, given the long duration of the emergency phase of such disaster, future studies are needed to document further its long-term effects.

This meta-analysis suffers from some limitations. First, the included studies were characterized by a high heterogeneity. For example, a wide variety of instruments has been used to measure the presence of psychological disorders and/or symptoms. Second, the number of studies that measured PTSD was relatively small. At the moment we are still in the acute phase of the pandemic, while the literature claims that PTSD occurs more in the post-disaster phases. Future research should focus on the medium and long-term consequences of the pandemic on both mental health and psychological wellbeing in children and adolescents. Third, we did not examine the effects of moderators such as age and sex. However, most of the studies did not examine differences by age group and/or sex. Fourth, the studies included in this meta-analysis did not represent all the continents. Fifth, we considered in our meta-analysis only studies indexed in PubMed, PsycINFO, and Scopus, and this could have limited and/or biased our results: future meta-analyses should widen the search also to other databases (Lemeshow et al., 2005). Sixth, we did not take into account whether the studies that we included in this meta-analysis were longitudinal or not, and this did not permit to systematically examine whether there was an increase in psychological disorders/symptoms due to the COVID-19 pandemic. Future research could consider further this issue. Seventh, given to the reduced time period in which we could search for studies about the COVID-19 pandemic, the number of eligible articles for our meta-analysis was quite small, as anticipated. However, the pandemic emergence is unluckily still ongoing, so researchers are continuing to publish studies about the topics that we examined. Basing on a larger number of studies, future systematic or meta-analytic reviews about the same topic could explore the moderating role of further characteristics of the studies concerning aspects related to samples, instruments, and designs.

This study documents some of the negative consequences of the current pandemic on children and adolescents’ mental health after about one year and a half from its outbreak. Based on our findings, we could argue that the pandemic urges not only the adoption of measures aimed at protecting physical health, but also psychological interventions to help children and adolescents coping with it. Young people have a large variety of resources that enable them, for example, to benefit from information and activities fostering their knowledge about the characteristics of a disaster, the protective behaviors, the associated emotions, and the emotional strategies deputed to manage them (Raccanello et al., 2020a, 2020c). However, our results can be viewed focusing on the bright side of the medal: about 80% of the participants did not show the disorders and/or symptoms at issue. Future research should further explore the factors underlying the occurrence of mental health disturbances in certain cases and the absence of their development in many other cases. Such knowledge would be of primary relevance to implement actions to sustain children and adolescents’ resilience, also for possible future traumatic events.
